# Interventional Effect of Nanosilver Paint on Fungal Load of Indoor Air in a Hospital Ward

**DOI:** 10.1155/2021/8658600

**Published:** 2021-12-20

**Authors:** Nasrin Rostami, Hossein Alidadi, Hossein Zarrinfar, Damon Ketabi, Hamed Tabesh

**Affiliations:** ^1^PhD Student of Environmental Health Engineering, School of Health, Tehran University of Medical Sciences, Tehran, Iran; ^2^Environmental Health Department, Health Sciences Research Center, School of Health, Mashhad University of Medical Sciences, Mashhad, Iran; ^3^Allergy Research Center, Mashhad University of Medical Sciences, Mashhad, Iran; ^4^Department of Occupational Health Engineering, School of Health, Mashhad University of Medical Sciences, Mashhad, Iran; ^5^Occupational Health Department, Health Sciences Research Center, School of Health, Mashhad University of Medical Sciences, Mashhad, Iran

## Abstract

Hospital ward environments contain various types of microorganisms, in which fungal agents are one of the main contaminants that may cause hospital-acquired infections. Regarding this, the aim of the present study was to evaluate the effect of nanosilver paint on reducing fungal contaminants of indoor air in an educational, research, and treatment center. Two rooms in the hematology ward were selected. One room was painted using usual paint (control room) and the other room was painted with paint containing nanosilver particles (experimental room). One hundred and twelve samples were collected using active (Anderson BioSampler) and passive (settle plate or open plate) air sampling techniques. The samples were incubated for 3–7 days at 35°C, and the positive fungal cultures were examined according to morphological and microscopic characteristics. Following active sampling, the mean and standard deviation of the number of colony-forming units (CFU/m^3^) of fungi colonies in the experimental and control rooms were 29.21 ± 17.99 and 22.50 ± 10.02 before intervention and 13.79 ± 6.20 and 31.07 ± 21.1 after intervention, respectively. Following passive sampling, the number of CFU/plate in the experimental and control rooms was 6 and 0 before and 1and 1 after intervention, respectively. The use of the nanosilver paint was effective in reducing air fungal contamination. Moreover, the active sampling method was more sensitive to measuring the concentration changes for fungal bioaerosols.

## 1. Introduction

Transmission of microbial pathogens through air is considered as an important way in indoor and outdoor environments [1]. High concentrations of microorganisms in air may be considered as an environmental risk, causing health problems in the indoor environments [2]. About 5–34% of indoor air pollution is caused by bioaerosols [3]. The results of epidemiological studies show that high concentrations of airborne microbes can be life-threatening and fatal [4]. Exposure to many microorganisms is inevitable in indoor environments such as hospitals [5]. It can be hazardous for hospitalized patients, especially those requiring long-term intensive care to exposure under the bioaerosols [6, 7]. Fungi are considered as one of the major sources of microbial contamination in indoor environments [1]. Fungal pollution of indoor environments depends on many factors such as temperature, moisture, ventilation, and organic matter present in building materials. Also, outdoor fungal spores may be transmitted through visitors, patients, and air conditioning [8]. Opportunistic fungal infections are usually reported in individuals with immune deficiency, such as hospitalized patients [7]. Therefore, fungal bioaerosols should be reduced in hospitals because of the high-risk groups; those may be sensitive or vulnerable to these dangerous biological agents [5]. The use of nanoparticles, including silver nanoparticles, is one of the ways that has been considered as a medical disinfectant in recent years [9]. The antifungal properties of silver nanoparticles have been reported in many studies by various researchers [10, 11]. Lee et al. concluded that silver nanoparticles have successful fungicidal activity against dermatophytes and pathogenic fungi and could be used as potential disinfectants against human fungal diseases [12]. Therefore, silver nanoparticles can be used as disinfectants due to their significant antimicrobial effect, along with a low toxicity and tolerance against different conditions. Although some nanoparticles clearly reduce the microbial load on various environments in laboratory settings, they have rarely been evaluated in well-designed clinical studies for their effectiveness in hospital environments.

Therefore, the purpose of this study was to evaluate the effect of nanosilver paint on reducing fungal contamination of indoor air in an educational, research, and treatment center, Mashhad, northeast of Iran.

## 2. Methods

Ethical approval was obtained from the Ethics Committee of Mashhad University of Medical Sciences, Iran (number: IR.MUMS.REC.1393.45).

### 2.1. Study Design

An interventional study was conducted in hematology ward of Imam Reza Hospital in Mashhad, northeast of Iran. Two rooms of this ward were selected (an experimental and a control) and examined before and after intervention (in 2014). This ward was selected because of its special significance and sensitivity due to hospitalization of cancer patients undergoing chemotherapy. They suffer immunodeficiency disorders, which are prone to hospital-acquired infections. Another reason for selecting this ward was the limited presence of visitors, as an interventional factor affecting the result of the study.

### 2.2. Painting the Experimental and Control Rooms

In this study, both control and experimental rooms were similar in terms of area, ventilation system, temperature conditions, pressure, humidity, sunshine direction, number of hospitalized patients, medical/nonmedical equipment, and the cleanup of the preexistent coating. Experimental room was coated with paint containing silver nanoparticles and control room was coated with ordinary paint, without any damp stains. The temperature and humidity of the experimental and control rooms were measured using a barometer (model PHB-318, Canada).

### 2.3. Air Sampling

To measure the concentration level of the fungal bioaerosols in indoor air of the hospital rooms, air sampling was performed twice a day. In the morning (8-9 am) and in the evening (5-6 pm), 14 times before and 14 times after the intervention, using both active and passive sampling techniques (seven days before and seven days after painting the rooms). Moreover, passive and active sampling was carried out simultaneously.

### 2.4. Active Air Sampling

SKC BioSampler (the single-stage Anderson, model 200 Hole, The United States) with a BioLite air sampling pump were used for the active air sampling. However, the sampling pump was placed inside a bag fully in order to insulate sound effectively to ensure the well-being of the patients admitted in the rooms. In order to avoid any microbial contamination before the sampling, the BioSampler was completely disinfected using 70% ethanol according to the device catalog (catalog number: 9610-225) and then placed under a UV lamp [13]. During sampling, a 90 mm petri dishes containing the Sabouraud dextrose agar (SDA; Merk, Germany) medium supplemented with chloramphenicol (SC) was located in the BioSampler. The pump was set at a flow rate of 14.15 L/min for 10 minutes. The sampling circuit was done 150–120 cm away from the patient's respiratory tract and at a height of 150–100 cm from the floor.

### 2.5. Passive Air Sampling

Petri dishes containing SC media were also used in the passive sampling method. During the sampling, the door of the plates was opened and placed at a height of 100–150 cm for 20 minutes [8]. After sampling, the door of petri dishes was completely closed with a paper glue to prevent the entry of secondary contamination and then transferred to the laboratory through a cold box. During the sampling, the door and windows of the room were always closed, and the personnel movements were prevented.

### 2.6. Examination of Fungal Agents

The plates containing air samples were transferred to the laboratory and incubated for 3–7 days at 32°C. Then, the fungal colonies were counted and identified according to morphological and microscopic characteristics.

### 2.7. Statistical Analysis

The SPSS 16 software, independent samples *t*-test, paired samples *t*-test, chi-square test, and Fisher's exact test were used in the preintervention and the postintervention assessment, in order to statistically prove the effect of nanosilver paint on the reduction of fungal contamination.

## 3. Results

In this study, 112 air samples (56 for each active and passive technique) were collected from the hematology ward. The results of active air sampling were reported as fungal colony-forming units (CFU)/m^3^, and the results of passive sampling were reported as CFU/plate.

### 3.1. Results of Active Sampling

**Figure 1:**
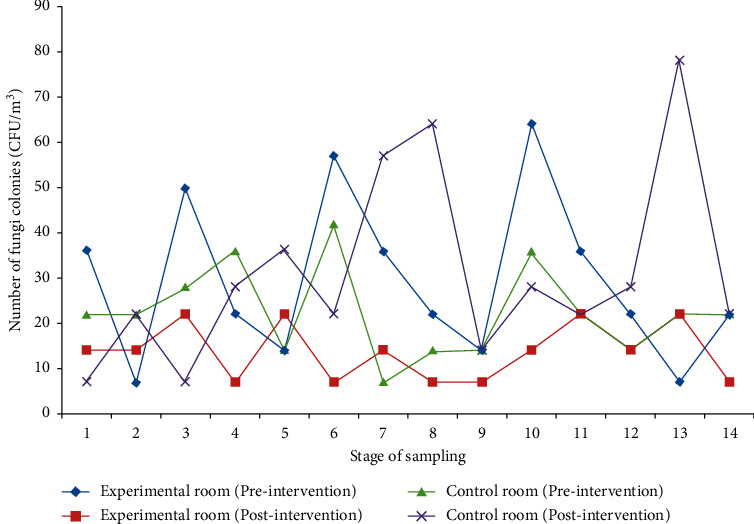
Comparison of changes in the number of colony-forming units (CFU/m^3^) of fungi colonies in terms of time in active sampling.

According to the results, both experimental and control rooms were contaminated by various fungal bioaerosols before intervention. However, there was not any significant difference between the air fungal load of the experimental and control rooms (*P* value = 0.237). The average numbers of fungal bioaerosols in experimental and control rooms were different after intervention (*P* value = 0.01). These results reflect to low concentration of fungal agents per cubic meter of air in the control room after the intervention. The mean and standard deviation of the number of fungal agents as CFU/m^3^ in the experimental room was 29.21 ± 17.99 before the intervention and 13.79 ± 6.20 after the intervention. This difference was significant with *P* value = 0.01 (Figure 1). On the other hand, the number of fungal agents per cubic meter of air was different before and after intervention in the experimental room. The mean and standard deviation of the number of fungal agents as CFU/m^3^ in the control room was 22.50 ± 10.02 before intervention and 31.07 ± 21.1 after intervention. This difference was not significant with *P* value = 0.24 (Table 1).

### 3.2. Results of Passive Sampling

The chi-square test and Fisher's exact test showed that the passive sampling data were often zero and one. The data analysis using these tests showed no significant difference between the preintervention stage (*P* value = 0.098) and the postintervention stage (*P* value = 1). This could indicate the major disadvantage of the passive sampling method compared to the active air sampling method for monitoring airborne fungal contamination.

### 3.3. Results of Environmental Conditions during Sampling

According to the results of room temperature measurement, the difference in temperature of the experimental and control rooms was not significant between the stages of preintervention (*P* value = 0.77) and postintervention (*P* value = 0.56). Moreover, the difference between the temperature of experimental room (*P* value = 0.57) and control room (*P* value = 0.05) was not significant before and after intervention. On the other hand, there was a similar temperature condition before and after intervention in both the experimental and control rooms (Table 2).

In addition, there was no significant difference between the humidity of rooms of the experimental and control rooms before (*P* value = 0.8) and after (*P* value = 0.92) intervention. The humidity difference between stages of preintervention and postintervention in the rooms of experimental (*P* value = 0.86) and control (*P* value = 0.53) was not significant either (Table 3).

## 4. Discussion

Indoor air of hospital wards could have a wide range of infectious microorganisms. Exposure to airborne microbes may have a more severe effect on susceptible groups, especially those who are immunocompromised [7]. Fungal agents are one of the most common microorganisms in hospital indoor environments, which can lead to nosocomial infections. The use of nanoparticles could be one of the ways that has been considered as a medical disinfectant in recent years [9]. In the current study, the use of the nanosilver paint was effective in reducing the air fungal contamination in an educational, research, and treatment center. According to the results, both the experimental and control rooms were contaminated by various fungal bioaerosols prior to the intervention. However, there was no significant difference between the fungal contamination load of the experimental and control rooms. The results also showed a significant difference in the fungal contamination between the preintervention and postintervention stages in the experimental room. Overall, these results indicate a reduction in the fungal contamination level in the experimental room air compared to the control room air.

Jabbari et al. compared the average number of fungi colonies in the experimental and control rooms and showed a significant difference between them. They showed the positive effect of nanosilver paint on reduction of air fungal pollution in hospitals [14]. However, Naddafi et al. showed that there is no significant difference between the average number of fungi colonies after use of nanosilver paint in rooms of experimental and control. Hence, their study did not indicate antimicrobial effectiveness of nanosilver paint to reduce the fungal contamination level in air [15]. According to Kaiser et al., the amount of nanosilver released from the painted surfaces over time is not so much as to give rise to toxic acute responses [16]. According to the results of Mueller and Nowak's study, the use of nanoparticles cause no adverse effects in the environment, and the concentration of silver nanoparticles in the environment is probably much lower than estimated values [17].

A very important point in interventional studies is that some possible factors can influence the actual outcome and its main objective. Therefore, the impact of other interventional factors should be reduced as possible. Although the two similar studies mentioned above have been carried out in different times and places, since both studies have used nanoparticle paint, other important parameters should also be similar. One of the influential factors in these studies is the air sampling method. In the two previous studies, despite the similarity of the objectives, air sampling methods were different. Jabbari's study was carried out using the passive sampling method, and Naddafi's study was performed using the active sampling method [14, 15].

Several studies have compared the results of active and passive sampling methods, and contradictory results have been obtained. Some studies have shown a significant difference between the results of these two sampling methods, while the results of other studies have shown that there is no difference between the results of the two methods [18]. Sudharsanam's study showed a significant difference between the results of active and passive sampling methods [19]. Napoli et al. also achieved a similar result in his investigation and found that there was a severe difference between the results of them [18]. However, in the present study, the active and passive sampling methods were used simultaneously for the experimental and control rooms in order to eliminate or reduce the impact of the sampling method. In fact, in accordance with the standards of air sampling methods, the active sampling could show a real level of fungal contamination.

Various studies conducted in the world on concentration of bioaerosols in hospitals proved that concentration of bioaerosols is different among various parts of a hospital as well as in different hospitals. For example, according to the studies by Ortize et al. in Spain and by Yassin and Almouqatea in Kuwait, different parts of each hospital vary in contamination load of airborne microorganisms [20, 21]. Hosseinzadeh et al. in his study in Hamadan concluded that the density and concentration of bioaerosols vary from hospital to hospital [22].

Therefore, it is necessary to reduce air fungal contamination load (AFCL) in various wards. On the other hand, according to the results of some studies, there is a significant relationship between the concentration of bioaerosols and environmental factors such as temperature and humidity [2, 5]. According to Bozic and Ilić' study, high humidity has greatly affected the concentration and growth of biological contaminants [23]. However, Jung et al. also showed that high temperature can have a negative effect on fungi [24]. For this reason, relative humidity and temperature of indoor air as interventional factors were examined in this study. According to the current results, there was no a significant difference in the temperature and humidity between the experimental and control rooms during the preintervention and postintervention stages. One of the limitations that can be mentioned in this plan includes the following: first, it was not possible to evaluate bacterial agents and was not examined in this regard. Second, the number of samples was limited, and if the number of samples was more, a stronger conclusion could be obtained. However, Naddafi et al. indicated that there was no statistically significant reduction in the bacterial agents between the control and case rooms and also between two types of paints by the active sampling method [15].

## 5. Conclusion

The results of the present study prove the effectiveness of paint containing silver nanoparticles in reducing airborne fungal contamination level in indoor air of hospitals. Therefore, the use of this kind of paint can reduce the concentration of fungal bioaerosols, especially in hospital wards with susceptible patients. However, the number of samples was limited, and a large sample size could improve data analysis and obtain a stronger conclusion.

## Figures and Tables

**Table 1:** Statistical analysis of air sampling data using the active method for fungal load of indoor air in a hospital ward.

Variable	Experimental room	Control room	Independent samples test
	Mean ± SD	Mean ± SD
Preintervention contamination	29.21 ± 17.99	22.50 ± 10.02	*P* value = 0.23
Postintervention contamination	13.79 ± 6.20	31.07 ± 21.1	*P* value = 0.01
Paired samples test	*P* value = 0.01	*P* value = 0.24	

**Table 2:** Statistical comparison of environmental temperature on fungal load of indoor air in a hospital ward.

Variable	Experimental room	Control room	Independent samples test
	Mean ± SD	Mean ± SD
Preintervention	27.32 ± 1.38	27.55 ± 1.62	*P* value = 0.77
Postintervention	27.07 ± 1.62	26.65 ± 0.86	*P* value = 0.56
Paired samples test	*P* value = 0.57	*P* value = 0.05	

**Table 3:** Statistical comparison of environmental humidity on fungal load of indoor air in a hospital ward.

Variable	Experimental room	Control room	Independent samples test
	Mean ± SD	Mean ± SD
Preintervention	31.84 ± 6.42	32.71 ± 6.14	*P* value = 0.80
Postintervention	31.35 ± 5.75	31.6 ± 3.63	*P* value = 0.92
Paired samples test	*P* value = 0.86	*P* value = 0.53	

## Data Availability

The data used to support the findings of this study are available from the corresponding author upon request.

## Abbreviations

CFU: Colony-forming units.
